# Differential Cellular Response to Mercury in Non-Farmed Fish Species Based on Mitochondrial DNA Copy Number Variation Analysis

**DOI:** 10.3390/biology13090691

**Published:** 2024-09-03

**Authors:** Marta Giuga, Venera Ferrito, Giada Santa Calogero, Anna Traina, Maria Bonsignore, Mario Sprovieri, Anna Maria Pappalardo

**Affiliations:** 1 Department of Biological, Geological and Environmental Sciences, Section of Animal Biology “M. La Greca”, University of Catania, Via Androne 81, 95124 Catania, Italy; vferrito@unict.it (V.F.); giada.calogero@phd.unict.it (G.S.C.); 2 National Research Council of Italy, Institute of Anthropic Impacts and Sustainability in Marine Environment (CNR-IAS), Via De Marini 16, 16149 Genova, Italy; 3 National Research Council of Italy, Institute of Anthropic Impacts and Sustainability in Marine Environment (CNR-IAS), Lungomare Cristoforo Colombo 452, 90149 Palermo, Italy; anna.traina@cnr.it; 4 National Research Council of Italy, Institute of Anthropic Impacts and Sustainability in Marine Environment (CNR-IAS), Via del Mare, 91021 Campobello di Mazara, Italy; maria.bonsignore@cnr.it; 5 National Research Council of Italy, Institute of Marine Sciences (ISMAR-CNR), Tesa 104—Arsenale, Castello 2737/F, 30122 Venezia, Italy; mario.sprovieri@cnr.it

**Keywords:** mtDNAcn, biomarker, teleost, marine monitoring, environmental contaminants, early warning

## Abstract

**Simple Summary:**

Mercury represent a serious threat for marine ecosystems due to its persistence in the environment. Fishes are the most numerous and widely distributed group of vertebrates, living in the sea with different species often studied and used as bioindicators of the quality of aquatic systems being able to reflect even small changes in environmental parameters. Mitochondria are small cell organelles with their own DNA and the number of mitochondria within a cell is highly variable in different animal tissues, depending on metabolic requirements. Mitochondrial genome is vulnerable to reactive oxygen species (ROS) which in turn impair mitochondrial function. Therefore, the aim of the present study was the validation of the variation in the number of mitochondrial DNA copies (mtDNAcn) as biomarker of oxidative stress in aquatic environment. Three selected fish species were collected in Augusta Bay, a contaminated area remarkable by past Hg inputs, and in a control area (Marzamemi and Portopalo di Capo Passero), both in the South-East of Sicily. Based on the evidence found, the assessment of mtDNAcn variation emerges as a valid biomarker of oxidative stress deriving from contaminant exposure.

**Abstract:**

Mercury (Hg) pro-oxidant role on biological systems and its biogeochemical cycle represent a serious threat due to its persistence in marine environment. As the mitochondrial genome is exposed to reactive oxygen species (ROS), the aim of the present study is the validation of the variation in the number of mitochondrial DNA copies (mtDNAcn) as biomarker of oxidative stress in aquatic environment. During summer 2021, three selected fish species (*Mullus barbatus*, *Diplodus annularis* and *Pagellus erythrinus*) were collected in Augusta Bay, one of the most Mediterranean contaminated areas remarkable by past Hg inputs, and in a control area, both in the south-east of Sicily. The relative mtDNAcn was evaluated by qPCR on specimens of each species from both sites, characterized respectively by higher and lower Hg bioaccumulation. *M. barbatus* and *P. erythrinus* collected in Augusta showed a dramatic mtDNAcn reduction compared to their control groups while *D. annularis* showed an incredible mtDNAcn rising suggesting a higher resilience of this species. These results align with the mitochondrial dynamics of fission and fusion triggered by environmental toxicants. In conclusion, we suggest the implementation of the mtDNAcn variation as a valid tool for the early warning stress-related impacts in aquatic system.

## 1. Introduction

The Mediterranean Sea is subjected to the continuous input of numerous anthropogenic chemical compounds that pose serious threats to the balance of the entire marine ecosystem. Many studies, especially in recent decades, have focused on better understanding the toxicity of these compounds. Some contaminants, in fact, have bioaccumulation capacity in different animal tissues and this represents a hazard factor with consequences observable even long after the period of actual exposure. Contaminants can be also transferred through trophic chain through the “biomagnification” phenomenon [1]. Furthermore, it should be taken into account that many organic and/or inorganic compounds can induce severe impacts on living systems even at sub-lethal concentrations [2,3].

Osteichthyes, or bony fish, are the most numerous and widely distributed group of organisms within marine ecosystems, occupying all available ecological niches in such environments. Therefore, different species have often been studied and used as bioindicators of the quality of aquatic systems being able to reflect even small changes in environmental parameters [4]. In particular, fish are the group most exposed to the uptake of Hg through gills, skin and gut [5].

The detection of environmental contaminants relying on sensitive analytical methods, does not provide evidence on their actual or potential impact on exposed organisms. Therefore, the scientific community is focusing on the definition of biomarkers as possible primary responses of organisms at the cellular, molecular and biochemical level which are indicative of a physiological dysfunction [6] caused by a disease, a stress condition, or exposure to environmental contaminants. Thus, biomarkers are a predictive tool of the impact that environmental contaminants might have at the organism level and subsequently at the population, community and ecosystem level. In this context, a possible source of early molecular biomarker could be the mitochondrial genome (mtDNA), which has a particular vulnerability to oxidative stress induced by Reactive Oxygen Species (ROS) because it lacks histones and nucleotide excision repair mechanisms unlike to nuclear DNA (nDNA). However, mitochondria are the main site of ROS generation both endogenously, from cellular respiration, and exogenously, from pro-oxidant substances (such as heavy metals). For these reasons, mitochondrial DNA (mtDNA) is more prone to possible mutations which can in turn lead to the synthesis of functionally impaired respiratory chain subunits and respiratory chain dysfunction causing a further increase in ROS production [7]. Therefore, dysfunctional mtDNA could indicate potential exposure to xenobiotic.

The number of mitochondria within a cell is highly variable in different animal tissues, depending, for example, on energy requirements [8], vulnerability to oxidative stress [9], activation of pathways that determine mitochondrial fission and fusion events which regulate fundamental processes such as cell migration and division, embryogenesis, and are responsible of the onset of a variety of diseases [10]. An indirect estimate of the number of mitochondria in each cell is the mitochondrial DNA copy number (mtDNAcn) which is closely related to the well-being of mitochondria [11].

In this context, many studies have shown how the variation of mtDNAcn can be used as an efficient predictive biomarker for the onset of cellular dysfunction and a variety of pathological conditions such as diabetes, cancer, and neurodegenerative diseases in humans [12,13,14,15,16]. In particular, several studies show a positive correlation between mtDNAcn variation and carcinogenesis or occupational diseases [17]. On the other hand, because damaged mtDNA can be removed by autophagy [18], oxidative damage to the mitochondrial genome could also manifest itself through a significant decrease in mitochondrial mass and, consequently, in the copy number of the genome itself, with inevitable bioenergetic and cellular dysfunction [19,20].

Therefore, mtDNAcn variation could be a promising biomarker also for defining the impact of xenobiotics and environmental insults of various nature on animal systems although, to date, there are few studies on the topic, both with application on model [21,22,23] and non-model [24] organisms.

Based on these evidences, in the present study the mtDNAcn variation will be taken into consideration to better understand how environmental contamination affects the mitochondrial function in three fish: red mullet, *Mullus barbatus* (Linnaeus, 1758), annular seabream, *Diplodus annularis* (Linnaeus, 1758) and common pandora, *Pagellus erythrinus* (Linnaeus, 1758) commonly used in the biomonitoring of the marine environment to study the effects of various pollutants such as metals, polycyclic aromatic hydrocarbons, phthalates and microplastics [25,26,27,28].

## 2. Materials and Methods

### 2.1. Area of Study

The sampling areas were chosen in order to have specimens of the three target species (*M. barbatus*, *D. annularis* and *P. erythrinus)* both from the area with a higher level of pollution detected in the Augusta bay (declared Site of National Interest SNI) and from a control site (Portopalo di Capo Passero for *M. barbatus*, Marzamemi for *D. annularis* and *P. erythrinus*). Since the 1970s, Augusta Bay has gained recognition on a global scale as a polluted marine environment [29]. Beginning in the 1950s, industrial activity quickly expanded until the 1980s: in particular, a chlor-alkali plant based on Hg-cell technology was deemed to pose a serious environmental risk to the marine environment because it released Hg into the water until environmental regulations were adopted in Italy [30]. Therefore, mercury contamination represents the main issue for this area, considering that heavy metals can bioaccumulate in tissues of aquatic organisms and, by domino effects, be transported to humans through the food chain [31,32,33].

**Figure 1:**
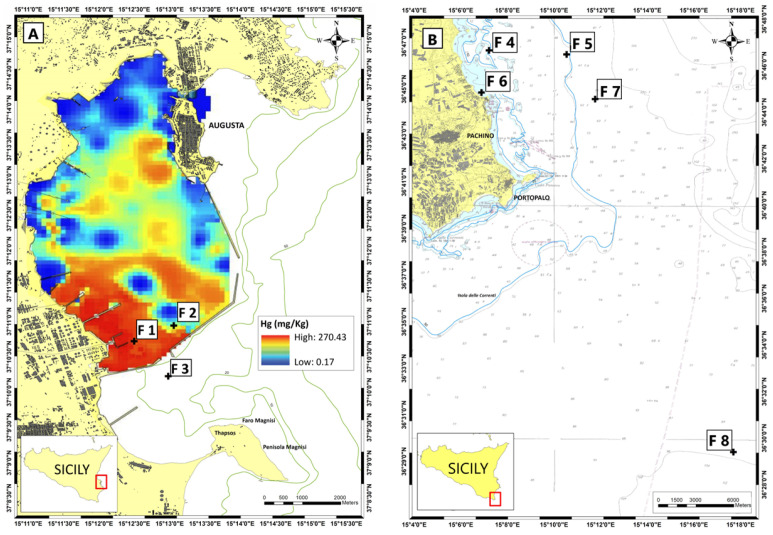
Sampling stations of fish (F1–F8) in the study area (**A**) shown on a map of the spatial distribution of Hg in sediments adapted from Sprovieri et al. [35] and in the control area (**B**) (adapted from [36]).

Two sites, Portopalo di Capo Passero and Marzamemi, both further south, were chosen as control sites. Indeed, according to the report on the biological and physico-chemical quality of marine coastal waters published in 2018 by the Regional Agency for Environmental Protection of Sicily [34], these sites were found to have good water and sediment quality. The sampling sites, which will be referred to hereafter as SNI (Augusta bay) and CTR (control sites), are shown in Figure 1.

### 2.2. Sampling

Fish sampling was carried out along the south-eastern Sicilian coast between July and early September 2021 through experimental fishing and the support of professional local fishermen.

The biota was collected in the southern section of Augusta Bay (collection stations F1, F2; Figure 1) since it shows higher levels of Hg both in the sediments and in the water column [35] and in the petrochemical district of Priolo (collection station F3; Figure 1) that is well-known to higher levels of Hg in sediments, too [37]. Two southern control areas were defined in front of Marzamemi (collection stations F4–F7; Figure 1) and off the south-east of Portopalo di Capo Passero (collection station F8; Figure 1) both declared in “good ecological and chemical quality status” [34].

In the SNI and off Marzamemi, the sampling was carried out using trammel net with length of 400–500 m (2–3 km in Marzamemi), height of 1.80 m and mesh size 25 of mm on sandy and gravelly bottoms with bathymetry between 10 and 18 m (10–50 m in Marzamemi). Both in the study and in the control area, fishing gears were lowered during the night, stationed for a few hours and retrieved at sunrise. Instead, the sampling of control specimens of *M. barbatus* took place about 12 miles south-east off Portopalo di Capo Passero through a trawl net set on a muddy seabed with bathymetry of 90–100 m. The net had a total length of 78 m, length of the codend of 8 m, vertical and horizontal opening of the mouth of 50 and 70 m respectively; initial mesh of the body of the net 80 mm; diagonal type codend mesh with a diameter of 50 mm. Once the presence of a shoal was detected with the aid of an echo-sounder, the net was lowered, towed for approximately 2 miles with a course 180° to the south at a speed of 3 mph, and then hoisted on board.

After collection, all fish specimens were immediately sacrificed by an overdose of MS222 (100 mg/L, Sandoz), according to [38], then were stored with flake ice and landed as quickly as possible. Once in laboratory morphological recognition of fishes was carried out following the analytical keys reported by [39], biometric parameters (total length, standard length and weight) were measured and a 1-cm fragment of muscular caudal fin was taken from each specimen, with sterile instruments in order to avoid contamination, and then stored in 96% ethanol before further processing.

The number of specimens of the three selected species (*M. barbatus, D. annularis* and *P. erythrinus*) from the study area and control area is shown in Table 1.

### 2.3. Chemical Analysis and Mercury Bioaccumulation

The total mercury concentration in fish species was measured using a direct mercury analyzer (Milestone DMA-80 atomic absorption spectrophotometry. Milestone, Bergamo, Italy). About 100 mg of wet sample (fish muscle of each specimen) were loaded into specific nickel boats and analyzed following the US-EPA 7473 method [40]. A Reference Standard Material (TORT-2 Lobster Hepatopancreas) was analyzed to assess accuracy (% recovery = 91–105%) and precision (routinely better than 8%; RSD%, *n* = 5).

### 2.4. DNA Extraction

Total DNA extraction was performed using the DNeasy Blood & Tissue Kit (Qiagen, Hilden, Germany) following the manufacturer’s protocol. Each DNA sample was then analyzed using the Nanodrop ND-1000 spectrophotometer (Thermo Fisher Scientific, Waltham, MA, USA) in order to quantify the DNA concentration: measurements of 260/280 nm and 260/230 nm absorbance ratios were used to determine the quality and degree of purity.

### 2.5. mtDNAcn Evaluation

Quantitative real-time PCR (qPCR) analysis using the comparative Ct method were performed with the QuantStudio 1 Real-Time PCR System (Thermo Fisher Scientific, Walthman, MA, USA) to estimate the relative mtDNAcn. At this aim, the mitochondrial *Cytb* gene was selected as target gene while the nuclear *18S* gene was used as reference gene (Table 2).

In each reaction 250 ng of total DNA were used in 20 µL of final volume containing 10 µL of SensiFAST SYBR master mix (Meridian BIOSCIENCE, Cincinnati, OH, USA) with ROX reference dye at low concentration (INVITROGEN, Waltham, MA, USA). Each set of reactions included a no template control and three technical replicates. qPCR conditions were performed following [24] experimental setup: 95 °C for 3 min, followed by 40 cycles of 95 °C for 20 s and 60 °C for 20 s. A final step of 95 °C for 15 s, 60 °C for 1 min and 95 °C for 1 s was included. Melt curves analysis were performed to detect non-specific PCR products.

Relative mtDNAcn for each sample was estimated according to Hartmann [43] and Rooney [44] equation: where cycle threshold (Ct) values of mitochondrial PCR products (mtDNA) were normalized to Ct-values of the nuclear locus (nDNA).

relative mtDNAcn = 2 × 2^ΔCt^

where ΔCt = Ct (nDNA gene) − Ct (mtDNA gene)

The variation in mtDNAcn in each fish species was obtained from the ratio between the mean of relative mtDNAcn value observed in the study area specimens and that exhibited by specimens of the same species taken in the control area. Finally, basing on the level of Hg bioaccumulation detected in each species collected in the SNI, when possible, the mtDNAcn variation will be assessed in fish showing medio-high level and high level of Hg bioaccumulation.

### 2.6. Statistical Analysis

All data obtained were subjected to statistical analysis using GraphPad Prism version 8.3.0. for Windows (GraphPad Software, San Diego, CA, USA, www.graphpad.com). The Mann-Whitney test was conducted to test the significance of the differences in mean observed between the two groups of specimens (polluted and control site samples) for *M. barbatus* and *P. erythrinus*, accepting those with *p* < 0.05 as significant values. For *D. annularis* statistically significant differences between the group SNI high Hg fish level, the group SNI medium-high Hg bioaccumulation fish level and the CTR group, was conducted thought the Tukey’s multiple comparisons test, accepting those with *p* < 0.05.

## 3. Results

**Figure 2:**
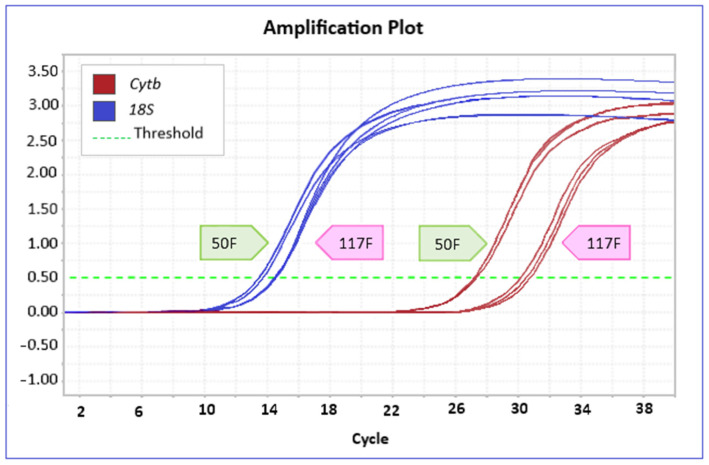
qPCR amplification plot comparing, in triplicate, 2 samples of *D. annularis*: one taken in the SIN (50F) and the other in Marzamemi (117F). The plot shows the fluorescence rate of the SYBR Green dye, denoted as ΔRn, versus the number of PCR cycles for the two targets: *Cytb* (red curve) and *18S* (blue curve). A lower Ct represents a higher DNA copy number in the initial sample. In the example shown, a higher mtDNAcn level is observed in the 50F sample from the contaminated site.

Determination of mtDNAcn performed by qPCR amplification showed consistently higher Ct values for the mitochondrial target gene (*Cytb*) than for the nuclear target gene, which corresponds to a lower amplification rate. This result aligns with the fact that the *18S* gene, used as the nuclear reference gene for calculating the relative mtDNAcn, is a tandemly repeated locus, so it shows higher amplification rates. Notably then, while Ct values for the nuclear gene were similar in specimens from both SNI and CTR sites in all species investigated, those for the mitochondrial target were very different in the two groups of organisms from the same species (Figure 2).

**Figure 3:**
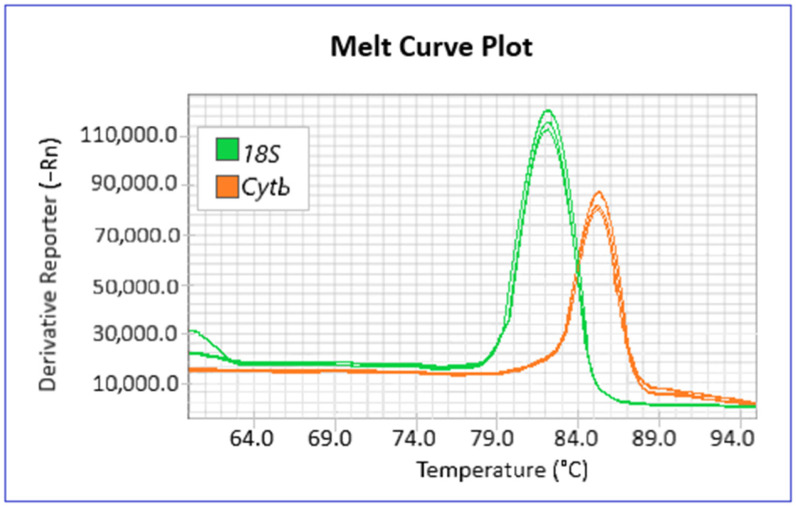
Melting curves of the *18S* gene (green curve) and *Cytb* target (orange curve) for a triplicate fish sample. The absence of nonspecific amplification products is shown.

Since the variation of mtDNAcn between the two survey areas is given by the ratio of the relative copy number in each, a higher value of this ratio in the contaminated site will result in an increase of mtDNAcn in the SNI compared CTR. Conversely, a lower relative mtDNAcn in the SNI will correspond to a decrease in mtDNA compared to CTR. Analysis of the melting curves shows specific amplification products (Figure 3).

**Figure 4:**
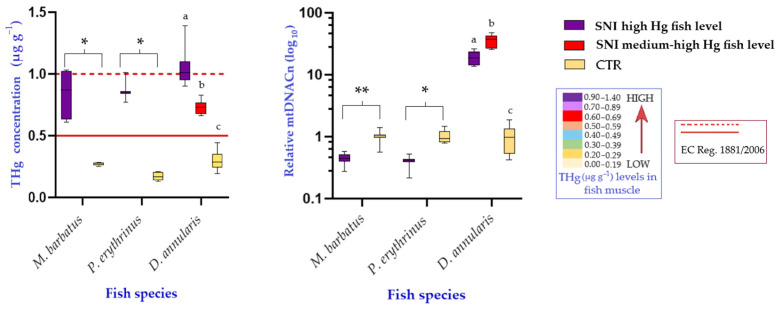
Comparative summary of the results in the three selected species from contaminated (SNI) and control (CTR) areas: total mercury (THg) bioaccumulation plots in fish muscle (**left**) and respective mtDNAcn variation (**right**). Different boxplots colors define the corresponding Hg level in biota. For *M. barbatus* and *P. erythrinus* statistically significant differences between SNI and CTR (Mann-Whitney) are denoted using * (*p* < 0.05) or ** (*p* < 0.005). For *D. annularis* statistically significant differences (*p* < 0.001) between SNI high Hg fish level, SNI medium-high Hg fish level and CTR (Tukey’s multiple comparisons test) are denoted using different letters. The figure reports also the admitted Hg level in fish for human consumption by the EC Reg. 1881/2006 (*M. barbatus* and *P. erythrinus*: dotted red line; *D. annularis*: continuous red line).

Analysis of the variation of mtDNAcn among the SNI specimens belonging to *M. barbatus* and *P. erythrinus* compared with their respective CTR shows a drastic reduction of the biomarker by 55% and 61%, respectively. The differences in mean values of mtDNAcn observed in the two groups (SNI vs. CTR) are statistically significant in both species with *p* < 0.005 in *M. barbatus* and *p* < 0.05 in *P. erythrinus*. It is noteworthy that, for both species in the study area, the bioaccumulation contents of Hg in muscle of SNI specimens, although significantly higher than those detected in the CTR specimens do not exceed the legal threshold of 1 µg/g fixed by the European Community Regulation (EC Reg.) 1881/2006, for the species under consideration (Figure 4). In *D. annularis* the legal threshold of bioaccumulation contents of Hg in muscle is set at 0.5 µg/g by EC Reg. 1881/2006. For this species two subgroups of specimens from SNI were analyzed having Hg levels above the legal threshold: the first subgroup with very high bioaccumulation content, comparable to those detected in *M. barbatus* and *P. erythrinus* and a second subgroup with medium to high levels of bioaccumulation.

Comparison between SNI and CTR specimens reveals a dramatic increase in mtDNAcn in the former. Specifically, in the first subgroup, an increase in copy number of about 20 times the average value observed in the CTR specimens is observed; in the second subgroup, the ratio of mtDNAcn is as much as 35 times higher in SNI than CTR. In both cases, the Mann-Whitney test reported statistically significant differences with *p* < 0.005 (Figure 4).

## 4. Discussion

The results obtained in the three target species (*M. barbatus, P. erythrinus* and *D. annularis*), indicate the existence of statistically significant variation in mitochondrial genome copy number as a cellular response to chronic exposure to chemical contaminants present in a highly impacted area such as the Augusta bay [30,37,45].

Fish species, in general, are particularly useful for assessing health status in the aquatic environment given their sensitivity to anthropogenic contaminants [33,46]. The choice of investigating the three mentioned species stems, on the one hand, from their commercial importance in Southeastern Sicily, which configures them as possible vectors of transfer of contaminants from the environment to humans through food; on the other hand, from the evaluation of their respective biological characteristics that place them in different ecological niches and trophic levels [47]. Such peculiarities could allow a better understanding of the mechanisms of contaminant transfer in biota (not considered in this study) and a more careful and integrated assessment of biological responses.

From the analysis of copy number variation carried out in the present study, apparently contrasting results emerge in the three target species. Specifically, in the specimens of *M. barbatus* and *P. erythrinus* from SNI, a significant reduction in the mitochondrial biomarker is observed compared with those from CTR, while *D. annularis* from SNI shows a significant increase in mtDNAcn. These results are not unexpected as both cellular responses are plausible. In fact, they appear to be compatible with the processes of mitochondrial fission and fusion, i.e., processes that lead, respectively, to the division of a mitochondrion into two smaller organelles and the union of two mitochondria into a larger one [48,49,50]. An increase in mitochondrial copy number occurs to accommodate the increased energy demand required to restore a condition of homeostasis; conversely, a decrease in mitochondrial genome copy number occurs when, at the cellular level, the system fails to compensate for the ongoing damage and mitophagy pathways are consequently activated in order to eliminate any damaged mitochondria [51,52].

More specifically, damage to mtDNA induces excessive ROS production through alteration of electron transport chain components, which in turn generates additional ROS fueling a vicious cycle. Such damage initially promotes mitochondrial genome replication, and thus an increase in mtDNAcn, sustained by mitochondrial fission events as a compensatory mechanism to increase energy production and remove damage to the mitogenome. However, following prolonged exposure to stressors, there is such an accumulation of mutations to mtDNA that this compensatory mechanism is irreversibly impaired, copy number decreases, and, as a result, mitochondria become dysfunctional by undergoing mitophagy [53,54,55].

It should be noted that a recent investigation [24] to a population of the sap-feeder insect, *Opsius heydeni,* from SNI the same contaminated area considered in this investigation, showed a significant decrease in mtDNAcn compared to specimens of the same species taken from a control site.

Several model organisms, from the nematode *Caenorhabditis elegans* [56] to higher organisms (mice, hamster), have shown both in vitro and in vivo, an increase in mtDNAcn after exposure to infrared radiation [57,58,59]. Among terrestrial invertebrates, change in mtDNAcn, as a biomarker of mitochondrial dysfunction, has been studied, for example, in the fruit fly *Drosophila melanogaster* in response to exposure to ochratoxin A. Again, exposure to the pro-oxidant agent resulted in an increase in mtDNAcn [21].

In the aquatic environment, the implementation of mtDNAcn variation for environmental monitoring has been validated on different animal groups. Kim et al. observed that exposure to polycyclic aromatic hydrocarbons (particularly fluoranthene and pyrene) resulted in a significant increase in mitochondrial genome copy number in the zebrafish *Danio rerio* [60].

In contrast, exposure to sub-lethal doses of the broad-spectrum insecticide bifenthrin in *Puntius sophore*, an edible fish species widespread in Asian freshwater, resulted in significant reduction of mtDNAcn in brain, liver, and muscle tissue [61]. In the sea turtle *Lepidochelys olivacea*, the change in copy number was related to the presence of a congenital malformation related to exposure to high levels of Hg. However, the investigation showed an apparent absence of association between the parameters investigated in the experimental groups [62]. Bowman et al., next determined mitogenome levels, in association with other cellular biomarkers (apoptosis and mutation rates in mtDNA), to define the effects of ultraviolet radiation on the skin of large marine mammals. The study showed a decrease in the mitochondrial genome in correlation with increased levels of UV-induced skin vesicles suggesting mtDNAcn variation as a valid biomarker of the effects of global warming in the marine environment [63]. Furthermore, the effects of exposure to environmentally important levels of dibutyl phthalate were investigates in the early life stage of zebrafish. High levels of oxidative stress damaging mitochondrial ultrastructure and function were observed which induce also the inhibition of mtDNAcn. In this case a downregulation of mRNA expression of mitochondrial fusion-related genes and an upregulation of fission-related genes were also detected [64].

Though studies on mtDNAcn variation in wildlife are scarce and often related to ageing [42,65,66], numerous investigations on the variation of this biomarker of oxidative stress induced by chemical, physical and environmental insults have been conducted on murine systems both in vitro and in vivo. In such model organisms, chronic exposure to chemical contaminants has shown, for example, a dose-dependent reduction between mtDNAcn and exposure to heavy metals [67] or ultrafine particulate matter [68]. In other cases, however, there has been an increase in mtDNAcn levels in response to exposure to secondhand smoke [69], infrared radiation [57] and proton beams [70]. Such evidence reinforces the theory that increased levels of mtDNAcn may help counteract mitochondrial dysfunction associated with increased oxidative stress, promoting, among others, the development of neurodegenerative diseases and the induction of carcinogenesis [70].

In some cases, the mtDNAcn levels can both increase and decrease within the same species in relation to, for example, the chronicity of the insult to which the organism is exposed. In this regard, a relationship between mtDNAcn and the effects of global warming was observed in the study by Li et al. in the white shrimp (*Palaemon carinicauda*). As water temperature and salinity increased, an initial increase in mtDNAcn was reported. However, a further increase in salinity induced a decrease in mtDNAcn consistent with irreversible impairment of mitochondrial function. In fact, ROS are generated during osmotic pressure regulation, which in turn induces oxidative stress. The authors therefore suggest that once the increase in salinity (and the simultaneous stimulation of mtDNA replication and ROS accumulation) exceeds a threshold value, mtDNA replication is disrupted due to polymerase blockade and, consequently, copy number is decreased [23]. The study by Syromyatnikov et al. reported the change in mtDNAcn as an effect of pesticide exposure in *Bombus terrestris*, one of the most important pollinators for agriculture, which is currently threatened with severe extinction risk mainly due to the intensive use of these xenobiotics. Interestingly, a different cellular response has been detected in relation to pesticide toxicity. Specifically, the action of systemic pesticides results in an increase in mtDNAcn, as a compensatory mechanism to the inhibition of mitochondrial respiration, while broad-spectrum pesticides act more heavily resulting in a significant decrease in the biomarker indicative, in turn, of irreversible mitochondrial dysfunction [71].

In the present study, as already described, at the same Hg bioaccumulation contents in the muscle tissue of specimens from the Augusta Bay, mtDNAcn levels are found to be halved for *M. barbatus* and *P. erythrinus*, while they are greatly increased in *D. annularis*. In this context, it is therefore necessary to clarify two aspects: (i) to interpret the differences found between the species analyzed and (ii) to identify a possible trend in the response observed in *D. annularis*. Regarding the first point, it is useful to consider the growth curves of the three species in order to highlight that *D. annularis* specimens being younger (about 1 year of age) than those of the other two target species sampled in the impacted area (3–4 years of age), but showing the same level of Hg bioaccumulation, probably exhibit a more efficient cellular response system towards damage from oxidative stress [42]. This response would be part of the compensatory mechanism of inhibition of cellular respiration resulting in increased mtDNAcn. In contrast, for *M. barbatus* and *P. erythrinus*, the longer exposure time to contaminants related to older age may have already impaired mitochondrial function resulting in a significant decrease in mtDNAcn. Taking the second point into consideration, it is useful to compare the mitochondrial copy number variation values observed in the *D. annularis* specimens with medium to high Hg levels with those detected in the group of specimens with high bioaccumulation values. It is observed that an even more pronounced increase in copy number is appreciated in the former than in the latter, confirming the trend of change in mtDNAcn [62], namely a rapid initial increase that undergoes a gradual decrease as bioaccumulation levels increase.

## 5. Conclusions

The present study represents one of the first investigation aimed at filling knowledge gap on the effect of mercury on mitochondrial stability in non-farmed fish species living in contaminated areas. To this end, a reproducible experimental setup was developed to detect mtDNAcn variation in three fish species characterized by high levels of bioaccumulation of Hg that is a potent pro-oxidant agent. The results obtained were interpreted considering that the primary cellular response to ROS generation is sustained by an increased mitogenome replication rate: in this case the increased mtDNAcn is suggested to be a valid early warning biomarker of contaminant exposure. Furthermore, it has been highlighted that upon chronic exposure to the contaminant, albeit at sub-lethal doses, the compensatory mechanism is compromised resulting in a reduction of mtDNAcn: in this case the decreased mtDNAcn is suggested to be a possible biomarker of effect to chronic xenobiotic exposure in marine environments.

## Figures and Tables

**Table 1:** Sampling and ecological information, biometric data and level of mercury (Hg) biaccumulation in fish muscle of the selected species. SNI, Site on National Interest; CTR, control areas; s.d., standard deviation; w.w., wet weight.

Species	Marine Habitat	Sampling				Total Length (Mean ± s.d. in cm)	Weight (Mean ± s.d. in g)	Hg w.w. (Mean in μg g^−1^*)*
		N°	Area	Station	Fishing Gear			
*Mullus barbatus*	benthic	6	SNI	F 1-2-3	trammel net	19 ± 2.7	80.4 ± 31.5	0.84 ± 0.20
		9	CTR	F 8	trammel net	16.6 ± 1.5	48.7 ± 12.9	0.30 ± 0.12
*Pagellus erythrinus*	demersal	10	SNI	F 1-2-3	trammel net	19.4 ± 4.1	109.6 ± 64.7	0.61 ± 0.24
		8	CTR	F 4-5-6-7	trammel net	18.2 ± 2	108.2 ± 48.7	0.25 ± 0.10
*Diplodus annularis*	demersal	10	SNI	F 1-2-3	trammel net	12.7 ± 0.7	35.8 ± 5.6	0.84 ± 0.24
		10	CTR	F 4-5-6-7	trawl net	13.1 ± 1.5	40.7 ± 13	0.34 ± 0.11

**Table 2:** Sequences of the primers used for qPCR.

Gene	Sequence 5′–3′	T° of Annealing	References
*Cytb*	F: CCATCCAACATCTCAGCATGATGAAA R: CCCCTCAGAATGATATTTGTCCTCA	60 °C	[41]
*18S*	F: TGTGCCCTAGAGGTGAAATT R: GCAAATGCTTTCGTTTCG	60 °C	[42]

## Data Availability

The data presented in this study are available on request from the corresponding authors.
